# Proteoglycan 4 attenuates ischemic post-stroke hemorrhagic transformation and blood-brain barrier disruption

**DOI:** 10.1016/j.ibneur.2025.09.008

**Published:** 2025-09-21

**Authors:** Jing Yang, Ning Tang, Ruanxian Dai, Jiajie Chen, Zhaojiao Li, Fengwen Jiang, Shiyi Li, Qiang Meng

**Affiliations:** a Faculty of Life Science and Technology, Kunming University of Science and Technology, Kunming 650500, P.R. China; b Department of Neurology, The Affiliated Hospital of Kunming University of Science and Technology, The First People’s Hospital of Yunnan Province, Kunming, Yunnan 650032, P.R. China; c Medical School, Kunming University of Science and Technology, Kunming 650500, P.R. China; d College of Science, Minzu University of China, Beijing 100081, P.R. China

**Keywords:** Stroke, Hemorrhagic transformation, Blood-brain barrier, Proteoglycan 4

## Abstract

**Background:**

Proteoglycan 4 (PRG4) possesses biological characteristics of anti-inflammation, lubrication, anti-apoptosis, and immunomodulation, and regulates the homeostasis of articular cartilage, myocardium, brain, and skin tissues. Nevertheless, the function of PRG4 in post-stroke hemorrhagic transformation (HT) and blood-brain barrier (BBB) disruption is unknown.

**Methods:**

A post-stroke HT mouse model (MACO-HT) was constructed and subsequently treated with recombinant PRG4 by tail vein injection. Neurologic impairment in mice was evaluated using mNSS and Zea longa score. The effect of recombinant PRG4 on HT was assessed using HE and TTC staining and hemoglobin assays. The effect of recombinant PRG4 on BBB repair was evaluated using Evan's blue leakage, western blotting, and IF staining assays.

**Results:**

Recombinant PRG4 reduced mNSS and Zea longa scores in MACO-HT mice. Infarct and hemorrhage areas and hemoglobin level in brain tissues of MACO-HT mice were significantly diminished after treatment with recombinant PRG4. MACO-HT mice exhibited significant Evan's blue leakage, which was ameliorated by reconstituted PRG4. Moreover, recombinant PRG4 notably diminished the levels of TLR2, MMP-2, and MMP-9 proteins, and augmented the levels of Claudin-5, Occludin, and ZO-1 in the brain tissues of MACO-HT mice.

**Conclusion:**

Recombinant PRG4 ameliorated post-stroke neurological impairment, HT, and BBB disruption. This finding identifies a biological function for PRG4 in stroke and provides support for therapeutic strategies targeting HT and BBB injury.

## Graphical Abstract

**Figure:**
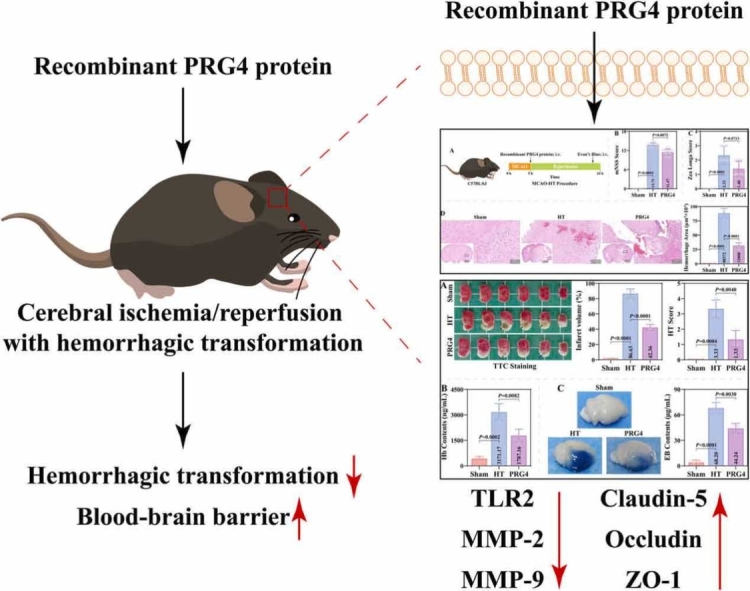

## 1 Introduction

Stroke possesses high morbidity, disability and mortality and is the third most prevalent contributor to global fatalities, superseded solely by coronary artery disorders and complications arising from SARS-CoV-2 infections (Hilkens et al., 2024, World Health Organization, 2024). More than 70 % of strokes are ischemic strokes (IS), even reaching 87 % in China. Intravenous thrombolysis (IVT) is currently the treatment strategy with the highest level of evidence and recommendation for early acute IS (AIS). However, only about 5 % of patients with AIS benefit from IVT due to the limitation of the therapeutic window (4.5 h), and 10 %-48 % of patients may be at risk of hemorrhagic transformation (HT) with increasing time (Tsivgoulis et al., 2023, Psychogios and Tsivgoulis, 2022). Mechanistically, reperfusion injury, neuroinflammation, and coagulation abnormality are the main causes of IVT-induced HT, especially BBB injury (Krag and Blauenfeldt, 2021, Charbonnier et al., 2020, Liu et al., 2023a, Li et al., 2024). During IVT treatment, rt-PA can contribute to MMP-2 and MMP-9 expression by binding to cell surface receptors such as protease-activated receptors, platelet-derived growth factor receptors, and lipoprotein receptors. MMP-2 and MMP-9 lead to BBB injury by specifically degrading tight junction proteins and extracellular matrix (ECM) between endothelial cells (ECs). Additionally, IVT causes apoptosis through activation of the caspase pathway and exacerbates the inflammatory cascade by activating leukocyte infiltration and microglia. These factors further exacerbate BBB damage and lead to HT. Therefore, it is crucial to prevent HT and improve the survival rate of AIS patients by in-depth study of the mechanism of HT-associated BBB injury and exploring effective therapeutic strategies.

ECs are one of the main functional units of the BBB, entangled with the ECM to form the endothelial glycocalyx layer (EGL), which is the brain's first line of defense against potentially harmful circulating substances, to maintain neurovascular homeostasis, substance transport, and BBB integrity (Logsdon et al., 2021, Baeten and Akassoglou, 2011, Zhao et al., 2021). EGL is a gel-like layer composed of glycosaminoglycans (GAGs) and proteoglycans (PGs). A variety of GAGs and PGs have been characterized as beneficial in maintaining BBB integrity after stroke, including perlecan, heparan sulfate, biglycan, and syndecan (Peck et al., 2023, Nakamura et al., 2019, Li et al., 2023, Smith and Melrose, 2024). Proteoglycan 4 (PRG4), alternatively designated as lubricin, represents a high-molecular-weight glycoprotein characterized by the covalent modification with chondroitin sulfate and keratan sulfate glycosaminoglycan chains (Ignatyeva et al., 2024, Saito, 2022). PRG4 was first found in the superficial zone of articular cartilage, where it acts as a lubricant in the joint cavity. Subsequent studies have confirmed that PRG4 is involved in the processes of osteoarthritis, hemorrhagic shock-induced cardiac injury, dry eye, and neoplasia by modulating inflammatory responses, mitochondrial function, oxidative stress, immune responses, fibrosis, and tissue regeneration (Takahata et al., 2022, Zhou et al., 2024, Menon et al., 2021, Oikawa et al., 2017). Additionally, recombinant PRG4 suppressed MMP activity and expression in synoviocytes and dry eye mice (Menon et al., 2021, Alquraini et al., 2017). It is suggested that the biological functions exerted by PRG4 are all related to BBB integrity. Notably, PRG4 alleviates traumatic brain injury by reducing neuroinflammation (Bennett et al., 2021). PRG4 produced by mesenchymal progenitor cells facilitates regeneration, repair and homeostasis of the dura mater (Mudigonda et al., 2022). Therefore, PRG4 may be a potent regulator in maintaining BBB integrity and preventing HT in stroke.

This study intends to construct an ischemic post-stroke HT (MCAO-HT) animal model to investigate the effect of recombinant PRG4 on HT and BBB integrity. The aim is to provide an experimental basis and new horizons for PRG4 as a potential target for ischemic post-stroke HT prevention and BBB repair.

## 2 Materials & methods

### 2.1 MCAO-HT procedure

This study was performed on male C57BL/6 J mice (18–22 g). C57BL/6 J mice had ad libitum access to autoclaved feed and reverse-osmosis purified water, as well as were housed in an SPF environment. Environmental parameters were rigorously controlled, including a 12-hour photoperiod regimen, stringently regulated thermal parameters at 23 ± 1°C, and humidity controls maintained within 55–65 %.

A total of 45 C57BL/6 J mice were randomly assigned to three groups—Sham, HT, and PRG4—with each group comprising 15 animals. As previously described (Tang et al., 2023, Jickling et al., 2014), mice in the HT and PRG4 groups were subjected to the MCAO-HT procedure. The right common carotid artery (CCA) of C57BL/6 J mice was surgically exposed after intraperitoneal injection of 3 % pentobarbital (0.1 mL/10 g) in C57BL/6 J mice. The proximal part of the CCA was ligated and the distal part of the CCA was clamped with an arterial clip. A live knot was tied to the CCA with a suture. Ophthalmic scissors were used to cut a notch from the bifurcation, and a thread plug containing sodium heparin was inserted into this notch. After releasing the arterial clip on the CCA, a filament was carefully advanced into the anterior cerebral artery. Mice in the PRG4 group were injected with 50 μg recombinant PRG4 (RPG246Mu01; Cloud-Clone, China) in the tail vein before reperfusion. The thread plug was removed after 5 h of cerebral ischemia to perform reperfusion to induce HT. Mice in the Sham group were only exposed to CCA without ischemia and reperfusion procedures. After 24 h of reperfusion, Zea longa and mNSS scores were performed in mice of each group to assess neurological impairment (Zausinger et al., 2000, Liu et al., 2023b).

### 2.2 TTC staining

The cerebellum and olfactory bulb were removed from the brains of mice in each group, and serial sections in the coronal plane were prepared (approximately 1 mm/slice). Brain sections were placed in 2 % TTC solution (17779; Millopore, USA) at 37 °C for 15 min to perform staining. Brain sections were then fixed in 4 % paraformaldehyde solution overnight. Next day, brain section images were collected and the cerebral infarct area was analyzed using Image J software. HT score based on TTC staining was performed using a double-blind method. HT was graded on a scale from 0 to 4, where 0 indicated no hemorrhage; 1, punctate HT at the infarct margin; 2, lamellar HT within the infarct core; 3, HT involving less than 30 % of the infarcted area; and 4, HT affecting 30 % or more of the infarcted region.

### 2.3 Evan’s blue leakage assay

Prior to euthanasia, mice in each group were injected in the tail vein with 2 % Evan's Blue (5 mL/kg; HY-B1102; MCE, USA). After 1 h, mice in each group were euthanized using an intraperitoneal injection of an overdose of pentobarbital containing lidocaine. The heart was perfused with saline until the blood was clear. Photographs were taken of the brains of each group to observe leakage of Evan's Blue. Brains were minced and placed in dimethylamide (1 mL/100 mg; C04255102; NJ-reagent, China). Following a 24-hour incubation at 60 °C, the brain tissue samples were centrifuged, and the supernatant was collected. A 96-well plate was prepared with sample, blank, and standard wells. Each well received 200 µL of brain tissue supernatant, dimethylamide, or varying concentrations of Evans Blue solution. Optical density was measured at 620 nm using a TGem Plus spectrophotometer (OSE-260–02; TIANGEN, Germany).

### 2.4 Tissue staining

HT area and expression of BBB markers (Claudin-5 and ZO-1) in brain tissues of each group were assessed using HE and IF staining. Fixed brain tissues were sequentially dehydrated, transparent, and embedded using ethanol, xylene, and paraffin, and 5 µm sections were prepared using a roller-type paraffin slicer (CUT 4062; SLEE, Germany). For HE staining, dewaxed brain sections were treated with distilled water, hematoxylin (E607317; Sangon, China), and eosin (E607321; Sangon) for 5 min, 5 min, and 1 min, respectively. For IF staining, dewaxed brain sections were subjected to antigenic repair and closure using sodium citrate repair solution (PR30001; Proteintech, USA) and goat serum (B900780; Proteintech). Brain sections were incubated sequentially with primary and secondary antibodies, including rabbit Claudin-5 pAb (1:150; PA5–99415; Invitrogen, USA), rabbit ZO-1 pAb (1:300; ab216880; Abcam, USA), and goat anti-rabbit IgG H&L (S0018; Affinity Biosciences, Australia). Imaging was performed using a Win digital slice scanning system (Winmedic, China) and a fluorescence microscope (DM3000 LED, Leica, Germany).

### 2.5 Western blotting assay

The protein extraction was performed by adding 400 μL of RIPA lysate (20–188; Millipore) to 100 mg of brain. After a boiling water bath, protein samples were analyzed for concentration using the “Protein” mode of an EzDrop 1000 spectrophotometer (Blue-Ray Biotech). Protein concentrations in the Sham, HT and PRG4 groups were (12.99 ± 2.27), (11.77 ± 1.43) and (12.47 ± 1.71) mg/mL, respectively. Protein samples (60 μg) were subjected to SDS-PAGE electrophoresis and subsequently transferred to 0.45 μm PVDF (FFP32; Beyotime, China). PVDF was blocked and incubated with skim milk powder (P0216; Beyotime) and antibodies, including rabbit β-actin pAb (1:2000; ab8227; Abcam), rabbit TLR2 mAb (1:1000; ab209216; Abcam), rabbit Claudin-5 pAb (1:1000; PA5–99415; Invitrogen), rabbit Occludin mAb (1:1000; ab216327; Abcam), rabbit ZO-1 pAb (1:1000; ab216880; Abcam, USA), rabbit MMP-2 pAb (1:2000; bs-0412R; Bioss, China), rabbit MMP-9 pAb (1:1000; ab283575; Abcam), and goat anti-rabbit IgG-HRP (ab6721; Abcam). A chemiluminescent gel imaging system (ChemStudio; Analytik Jena; Germany) was used for exposure and image collection of PVDF.

### 2.6 Statistical analysis

Data were expressed as mean±SD and statistically analyzed and visualized by GraphPad Prism software. Comparison of HT, mNSS and Zea longa scores was performed using the Kruskal-Wallis test. The remaining data were analyzed using one-way ANOVA. P < 0.05 represents a statistically significant difference.

## 3 Results

### 3.1 Recombinant PRG4 alleviates ischemic post-stroke HT

**Fig. 1:**
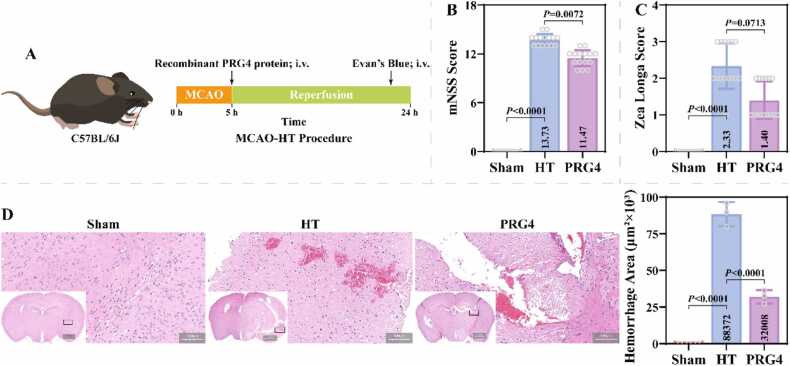
Effect of Recombinant PRG4 on neurological impairment and brain pathology in MCAO-HT mice. A: Flowchart of animal procedures and recombinant PRG4 treatment in this study. The i.v. stands for tail vein injection. B-C: The mNSS and Zea Longa scores were used to assess neurological impairments of mice in the Sham, HT, and PRG4 groups (n = 15; Kruskal-Wallis test). D: Pathological changes in the brain tissues of mice in Sham, HT and PRG4 groups were observed using HE staining and statistical analysis of hemorrhage area (n = 3; one-way ANOVA). Scale= 100 µm or 1000 µm.

**Fig. 2:**
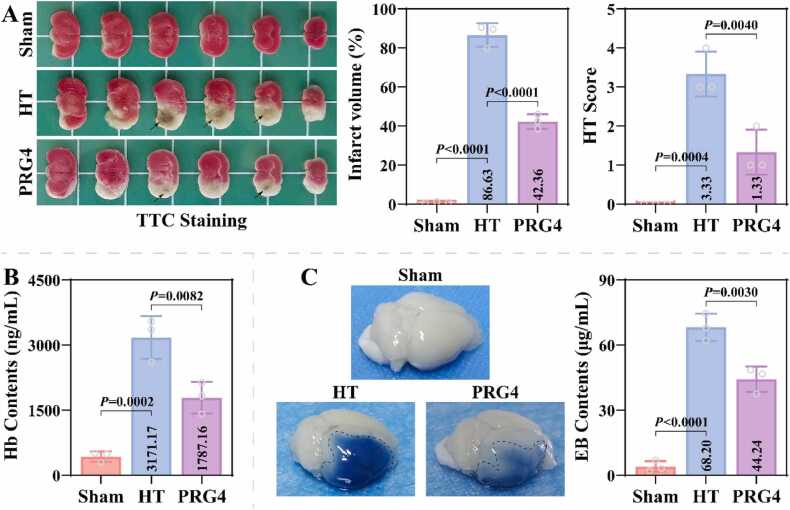
Effect of Recombinant PRG4 on cerebral infarction and hemorrhagic transformation (HT) in MCAO-HT mice. A: The cerebral infarct area and HT score of mice in Sham, HT and PRG4 groups were assessed using TTC staining (n = 3; Kruskal-Wallis test). B: The Hb content of mouse brain tissues from Sham, HT and PRG4 groups was detected using mouse hemoglobin (Hb) ELISA kit (n = 3; one-way ANOVA). C: Mice in the Sham, HT and PRG4 groups were injected with Evan's blue in the tail vein to assess the integrity of the blood-brain barrier (BBB) (n = 3; one-way ANOVA).

The animal procedures and recombinant PRG4 treatment for this study are shown in Fig. 1A. Compared to the Sham group, mice in the HT group exhibited a significant increase in mNSS scores (13.73 ± 0.68), as evidenced by limb flexion, rotation/tilting to the side of the hemiplegia, diminished sense and balance, and loss of reflexes (Fig. 1B). Additionally, mice in the HT group had significantly higher Zea Longa scores (2.33 ± 0.60) than those in the Sham group, as evidenced by rotation and inability to fully extend limbs and walk (Fig. 1C). Recombinant PRG4 treatment resulted in decreased mNSS (11.47 ± 0.96) and Zea Longa (1.40 ± 0.49) scores in HT model mice. HE staining showed that the brain tissue cells of Sham group were closely and evenly distributed with abundant cytoplasm and round nuclei (Fig. 1D). In the HT group, the vascular wall of the brain tissue was disrupted, and many erythrocytes in clusters were seen to leak into the extravascular brain parenchyma, with cells showing structurally lysed damage. Recombinant PRG4 treatment improved brain tissue damage and hemorrhage area in the HT group. Statistical analysis revealed that the hemorrhagic area of brain tissue was significantly higher in the HT group (88.37 ± 6.66) than in the Sham group, which was mitigated by recombinant PRG4 (32.00 ± 3.78). TTC staining indicated a significant increase in the infarct area of the brain tissue in the HT group (86.63 ± 4.98), which was ameliorated by recombinant PRG4 treatment (42.36 ± 3.10) (Fig. 2A). Additionally, compared to the Sham group, mice in the HT group had significantly increased HT scores (3.33 ± 0.47), as evidenced by punctate and lamellar hemorrhages within the infarct zone. Recombinant PRG4 treatment resulted in reduced HT score in MCAO-HT model mice (1.33 ± 0.47). Hb content in the brain was significantly higher in the HT group (3171.17 ± 404.34) than in the Sham group, and lower in the PRG4 group (1787.16 ± 300.21) than in the HT group (Fig. 2B). These results suggest that recombinant PRG4 effectively mitigated stroke-induced HT.

### 3.2 Recombinant PRG4 mitigates ischemic post-stroke BBB disruption

**Fig. 3:**
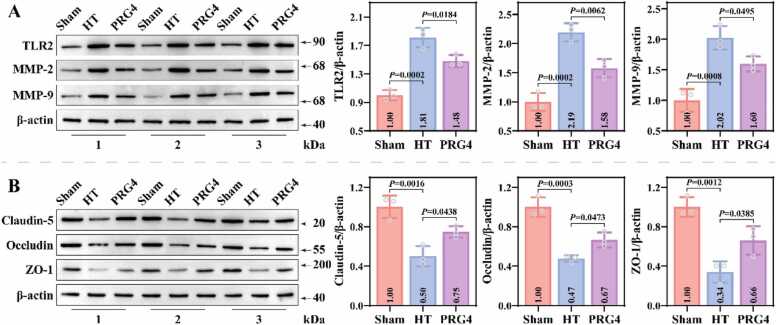
Effect of Recombinant PRG4 on BBB-related protein expression in MCAO-HT mice. A: The levels of TLR2, MMP-2 and MMP-9 proteins in brain tissues of Sham, HT and PRG4 groups were detected by western blotting assay (n = 3; one-way ANOVA). B: Representative images of gel blot of BBB-related proteins (Claudin-5, Occludin and ZO-1) and their differential expression analysis in Sham, HT and PRG4 groups (n = 3; one-way ANOVA).

**Fig. 4:**
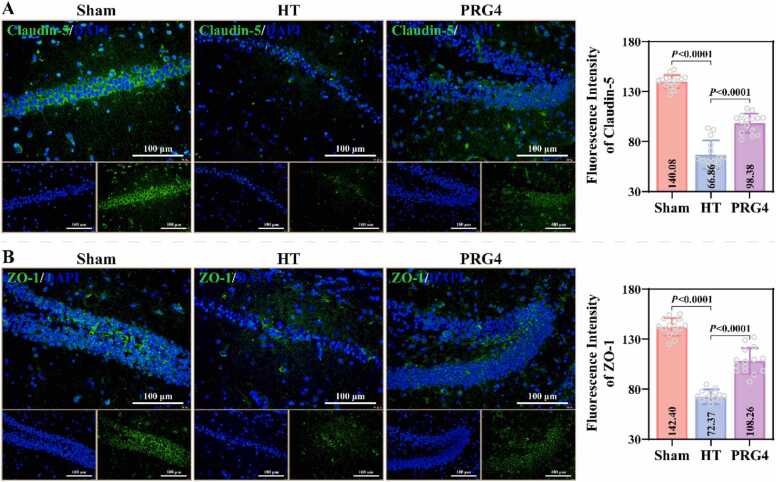
Effect of Recombinant PRG4 on Claudin-5 and ZO-1 levels in brain tissue of MCAO-HT mice. A-B: IF staining was used to observe the localization and fluorescence intensity of Claudin-5 (A) and ZO-1 (B) in brain tissues of Sham, HT and PRG4 groups (n = 3; one-way ANOVA). Scale= 100 µm.

The present study further evaluated the effect of recombinant PRG4 on BBB integrity in MCAO-HT mice. Evan's blue leakage assay revealed that the brains of the HT and PRG4 groups exhibited significant leakage of Evan's blue compared to the Sham group, and PRG4 group had less area and amount of Evan's blue leakage (Fig. 2C). Statistical analysis confirmed this result. Western blotting results revealed that the levels of TLR2 (1.81 ± 0.11), MMP-2 (2.19 ± 0.13), and MMP-9 (2.03 ± 0.16) proteins were significantly higher in the brain tissues of the HT group than those of the Sham group, and that the levels of these proteins were lower in PRG4 group (1.48 ± 0.07; 1.58 ± 0.13; 1.60 ± 0.10) than those of the HT group (Fig. 3A). Additionally, the levels of Claudin-5 (0.50 ± 0.08), Occludin (0.48 ± 0.03), and ZO-1 (X ± X) proteins were significantly lower in the brain tissues of the HT group compared to the Sham group; the levels of these proteins were significantly higher in the PRG4 group compared to the HT group (0.75 ± 0.05; 0.67 ± 0.06; 0.34 ± 0.09) (Fig. 3B). IF staining confirmed that Claudin-5 and ZO-1 proteins were mainly localized in the cell membrane, and the fluorescence intensity of Claudin-5 and ZO-1 proteins was significantly higher in both Sham and PRG4 groups than in HT group (Fig. 4A-4B). These results suggest that recombinant PRG4 effectively mitigates stroke-induced BBB damage.

## 4 Discussion

Impairment of BBB integrity is the main cause of IVT-induced HT in patients with AIS. The EGL composed of ECs and ECM is the main structure that maintains the integrity and function of the BBB. PRG4 belongs to the ECM and is associated with nerve repair, homeostasis and regeneration. However, the effect of PRG4 on HT and BBB damage in AIS is unknown. This study found for the first time that recombinant PRG4 alleviated neurological impairments, BBB damage, and HT in a stroke mouse model. The animal model chosen for this study was the MCAO-HT model without rt-PA. The relationship between HT induced by rt-PA and ischemia duration is still controversial (Copin and Gasche, 2008). Most studies set the time point of HT induction in subject animals with a treatment window (4.5 h) of rt-PA (Zhang et al., 2009, Wei et al., 2017). However, the pathological process in the ischemic hemianopsia is characterized by a spatiotemporal cascade of responses and there are differences between animals and humans. Additionally, the effects produced by rt-PA are difficult to distinguish from ischemia and reperfusion. Most importantly, the incidence of HT in the animal model was largely dependent on the duration of ischemia, which is consistent with clinical findings (Jickling et al., 2014, Lu et al., 2009). Previous studies have shown that the probability of HT in animals with MCAO lasting 1.5, 2.5, 3.5, and 5 h was 25 %, 50 %, 75 %, and 100 %, respectively, and was accompanied by increased mortality (Fagan et al., 2003). This is consistent with the clinical characterization of HT and death in patients with AIS. Clinically, ischemia for more than 6 h in patients with AIS is an independent predictor of the development of HT (Molina et al., 2001). These are the reasons why the MCAO-HT model was chosen for this study. The MCAO-HT model in this study was ischemic for 5 h and exhibited neurological impairments (increased mNSS and Zea Longa scores), HT (increased HT scores and Hb levels), and BBB injury (decreased Claudin-5, Occludin, and ZO-1 levels). This is consistent with the characteristics of post-stroke HT (Tang et al., 2023, Jickling et al., 2014, Shearer et al., 2018), implying that the development of the MCAO-HT model in this study was successful. Importantly, the present study found that recombinant PRG4 attenuated neurological impairment, HT and BBB disruption in the MCAO-HT model.

Mechanistically, this study found that recombinant PRG4 reduced the levels of TLR2, MMP-2 and MMP-9 in brain tissues of the MCAO-HT model. TLRs were the first to recognize important mediators of innate immune system activation. TLRs are expressed in glial cells and neurons of the central and peripheral nervous systems after brain injury, especially TLR2 and TLR4 (Tajalli-Nezhad et al., 2019, Lua et al., 2021). TLRs are able to interact with endogenous and exogenous molecules released during ischemia and may increase tissue damage. Previous studies have demonstrated that PRG4 reduced TLR2 activity in arthritis and traumatic brain injury (Bennett et al., 2021, Alquraini et al., 2015) and inhibited MMP-9 levels in dry eye and arthritis (Menon et al., 2021, Alquraini et al., 2017). This is consistent with the results of this study. Although the diseases are different. Notably, TLR2 facilitates BBB damage and HT, which is associated with the ONOO/HMGB1 and TLR4/MyD88 pathways (Chen et al., 2020, Wang et al., 2020). Furthermore, activation of the TLR2 pathway leads to increased levels of MMP-9 in AIS and cerebral hemorrhage (Min et al., 2015, Zhu et al., 2019). Numerous studies have confirmed that MMP-9 is highly expressed in the serum of AIS patients and predicts patient deterioration (Turner and Sharp, 2016, Lu and Wen, 2024). This is associated with increased cerebral infarct volume, in response to rt-PA, neurological deterioration, severe cerebral edema, HT, neuroinflammation, and BBB damage caused by MMP-9. This suggests that PRG4-alleviated neurologic impairment, HT and BBB disruption are associated with inhibition of the TLR2/MMP-2/MMP-9 pathway. After stroke, neutrophils are the first immune cells to release chemokines and cytokines in response to ischemic injury (Downes and Crack, 2010, Xu et al., 2021, Walz and Cayabyab, 2017). This is attributed to TLR2 through activation of NF-κB-producing chemokines such as CCL2 and CXCL2. MMP-9 is mainly derived from neutrophils and is able to lyse the ECM in the BBB, thereby allowing neutrophils to enter brain tissue. Interestingly, *Prg4*^-/-^ mice associated with gout and camptodactyly-arthropathy-coxa vara-pericarditis syndrome had increased neutrophil infiltration in the peritoneal cavity and peripheral blood, which was alleviated by recombinant PRG4 (Elsayed et al., 2021, Novince et al., 2011). Therefore, we suggest that recombinant PRG4 alleviates BBB injury from MMP-9 produced by neutrophils via inhibition of the TLR2-mediated NF-κB pathway, thereby suppressing post-stroke HT and neurological impairments.

Nevertheless, this study lacks studies related to recombinant PRG4-mediated neutrophil infiltration. Moreover, there is a lack of cellular experiments to validate the function of recombinant PRG4. All of this remains to be explored in the future. In conclusion, the present study demonstrated for the first time that recombinant PRG4 ameliorated post-stroke neurological impairment, HT and BBB disruption. It is suggested that PRG4, a novel TLR2/MMP regulatory protein, may serve as a potential new therapeutic agent for the treatment of post-stroke HT and BBB injury.

## CRediT authorship contribution statement

**Fengwen Jiang:** Conceptualization, Investigation, Methodology, Validation, Writing – review & editing. **Shiyi Li:** Conceptualization, Investigation, Methodology, Validation, Writing – review & editing. **Qiang Meng:** Conceptualization, Data curation, Funding acquisition, Project administration, Software, Supervision, Writing – review & editing. **Jing Yang:** Conceptualization, Data curation, Formal analysis, Funding acquisition, Investigation, Methodology, Project administration, Validation, Visualization, Writing – original draft. **Ning Tang:** Conceptualization, Data curation, Formal analysis, Funding acquisition, Investigation, Methodology, Project administration, Validation, Visualization, Writing – review & editing. **Ruanxian Dai:** Conceptualization, Formal analysis, Investigation, Methodology, Validation, Visualization, Writing – review & editing. **Jiajie Chen:** Conceptualization, Formal analysis, Investigation, Methodology, Resources, Validation, Writing – review & editing. **Zhaojiao Li:** Conceptualization, Investigation, Methodology, Software, Validation, Writing – review & editing.

## Ethics approval and consent to participate

The study was permitted by the Animal Ethics Committee of Kunming Medical University (kmmu20241922).

## Consent to publication

Not applicable.

## Funding

This work was supported by the Guangdong Brain Science Application Association Clinical Research Support Program for Young and Middle-aged Doctors in Neurology (BSAA2024-AD-01011), the Joint Fund of Yunnan Provincial Science and Technology and Kunming Medical University (202401AY070001-107), the Kunming University of Science and Technology Medical Joint Project (KUST-KH2022010Y), the Yunnan Provincial Clinical Medical Center for Spine and Spinal Cord Diseases Open Subjects (2024JZKFKT-23) and the 10.13039/501100001809National Natural Science Foundation of China (81960228).

## Conflicts of Interest

The authors declare that they have no known competing financial interests or personal relationships that could have appeared to influence the work reported in this paper.

## Data Availability

The data are available on request from the corresponding author.
